# Opportunities and barriers for telemedicine in pain management: insights from a SIAARTI survey among Italian pain physicians

**DOI:** 10.1186/s44158-024-00202-1

**Published:** 2024-09-17

**Authors:** Marco Cascella, Massimo Antonio Innamorato, Silvia Natoli, Valentina Bellini, Ornella Piazza, Roberto Pedone, Antonino Giarratano, Franco Marinangeli, Luca Miceli, Elena Giovanna Bignami, Alessandro Vittori

**Affiliations:** 1 Unit of Anesthesiology, Intensive Care Medicine, and Pain Medicine, Department of Medicine, Surgery, and Dentistry, University of Salerno, Baronissi, Salerno, 84081 Italy; 2 AUSL Romagna, Pain Unit, Department of Neuroscience, Santa Maria Delle Croci Hospital, Ravenna, 48121 Italy; 3 Department of Clinical-Surgical, Diagnostic and Pediatric Sciences, University of Pavia, Pavia, 27100 Italy; 4 Anesthesiology, Critical Care and Pain Medicine Division, Department of Medicine and Surgery, University of Parma, Parma, Italy; 5 Department of Psychology, University of Campania Luigi Vanvitelli, Caserta, 81100 Italy; 6 Department of Precision Medicine in Medical, Surgical and Critical Care (Me.Pre.C.C.), University of Palermo, Palermo, Italy; 7 Department of Anaesthesia, Intensive Care and Emergency, University Hospital Policlinico ‘Paolo Giaccone’, Palermo, Italy; 8 Department of Anesthesiology, Intensive Care and Pain Treatment, University of L’Aquila, L’Aquila, Coppito, 67100 Italy; 9 Department of Pain Medicine, IRCCS C.R.O. National Cancer Institute of Aviano, Pordenone, Aviano Italy; 10 Department of Anesthesia and Critical Care, ARCO ROMA, Ospedale Pediatrico Bambino Gesù IRCCS, Rome, 00165 Italy; 11 Pain Unit, Fondazione IRCCS Policlinico San Matteo, Pavia, Italy

**Keywords:** Telemedicine, Telehealth, Pain medicine, Artificial intelligence

## Abstract

**Background:**

The integration of telemedicine in pain management represents a significant advancement in healthcare delivery, offering opportunities to enhance patient access to specialized care, improve satisfaction, and streamline chronic pain management. Despite its growing adoption, there remains a lack of comprehensive data on its utilization in pain therapy, necessitating a deeper understanding of physicians’ perspectives, experiences, and challenges.

**Methods:**

A survey was conducted in Italy between January 2024 and May 2024. Specialist center members of the SIAARTI were sent an online questionnaire testing the state of the art of telemedicine for pain medicine.

**Results:**

One-hundred thirty-one centers across Italy reveal varied adoption rates, with 40% routinely using telemedicine. Regional disparities exist, with Northern Italy showing higher adoption rates. Barriers include the absence of protocols, resource constraints, and bureaucratic obstacles. Despite challenges, telemedicine has shown positive impacts on service delivery, with increased service volume reported. Technological capabilities, including image sharing and teleconsultation with specialists, indicate promising interdisciplinary potential.

**Conclusions:**

The integration of advanced telemedicine software utilizing artificial intelligence holds promise for enhancing telemonitoring and alert systems, potentially leading to more proactive and personalized pain management strategies.

## Background

The landscape of healthcare delivery has undergone a significant transformation with the implementation of telemedicine-based care pathways [1–3], even in the field of pain management [4–6]. Pieces of evidence suggest that telemedicine can offer the potential to enhance patient access to specialized care [7], improve patient satisfaction [8–10], and streamline the management of chronic pain [11]. Therefore, as the demand for effective pain management continues to rise, the integration of telemedicine into clinical practice has become increasingly relevant [12–14].

Nevertheless, despite its growing adoption, there remains a lack of comprehensive data on how telemedicine is being utilized by healthcare professionals specifically in pain therapy [15]. Consequently, understanding the perspectives, experiences, and challenges faced by physicians in implementing telemedicine for pain management is crucial for optimizing its use and addressing potential barriers.

This article presents the findings of a survey conducted by the Italian Society of Analgesia, Anesthesia, Resuscitation, and Intensive Care (SIAARTI) among pain physicians to assess the current usage, benefits, and limitations of telemedicine in pain management. The survey aimed to gather insights into physicians’ experiences, satisfaction levels, perceived effectiveness, and the impact of telemedicine on patient care in the context of pain therapy. The results of this analysis could provide valuable information that can inform future strategies for the integration of telemedicine in pain management practices.

## Methods

The survey was conducted across Italy between January 2024 and May 2024. All center members of the SIAARTI were asked to participate by an email invitation, which was sent to all directors of complex operative units, and by further advertising through the SIAARTI newsletter and its social media platforms. Participating centers were required to select one participant as representative physician of each given center for a computer-aided web interview (CAWI) using the free software SurveyMonkey. No specific exclusion/inclusion criteria were established as the scope of the survey is to provide an as-much-comprehensive-as possible overview of the Italian pain centers’ scenario about telemedicine for pain medicine. Answers were collected on an anonymous basis. A data clean procedure was also performed to remove duplicate answers within the same center. The survey was organized by SIAARTI and conducted in compliance with the EphMRA code of conduct. All participants in the survey provided voluntary, informed consent to data collection and use, based upon a clear understanding of the purpose of the data collection.

### Questionnaire and item assessment

The questionnaire comprised 33 questions addressing the following items: center organization, clinical activity, adoption of telemedicine, protocols for telemedicine, and clinical governance. This questionnaire was not an international and validated one, since it was created ad hoc for the Italian reality and with an exploratory intent.

### Statistical analysis

Descriptive statistics were employed to provide a comprehensive overview of the dataset. The primary focus was on calculating the absolute and percentage frequencies for the variables under consideration. Absolute frequencies were used to count the occurrences of each category within the variables, providing a straightforward measure of distribution. Complementing this, percentage frequencies were calculated to offer a relative measure, thereby enabling easier comparison between different categories regardless of their size. This dual approach of utilizing both absolute and percentage frequencies facilitated a thorough understanding of the data distribution, revealing patterns and trends that are critical for further interpretation and discussion.

## Results

A survey on the utilization of telemedicine in pain therapy was conducted, with responses collected from 131 centers across different Italian regions. From our previous cross-sectional analysis, we calculated that in Italy, there were 305 active pain centers; therefore, we have a 43.52% of respondents [16].

**Fig. 1:**
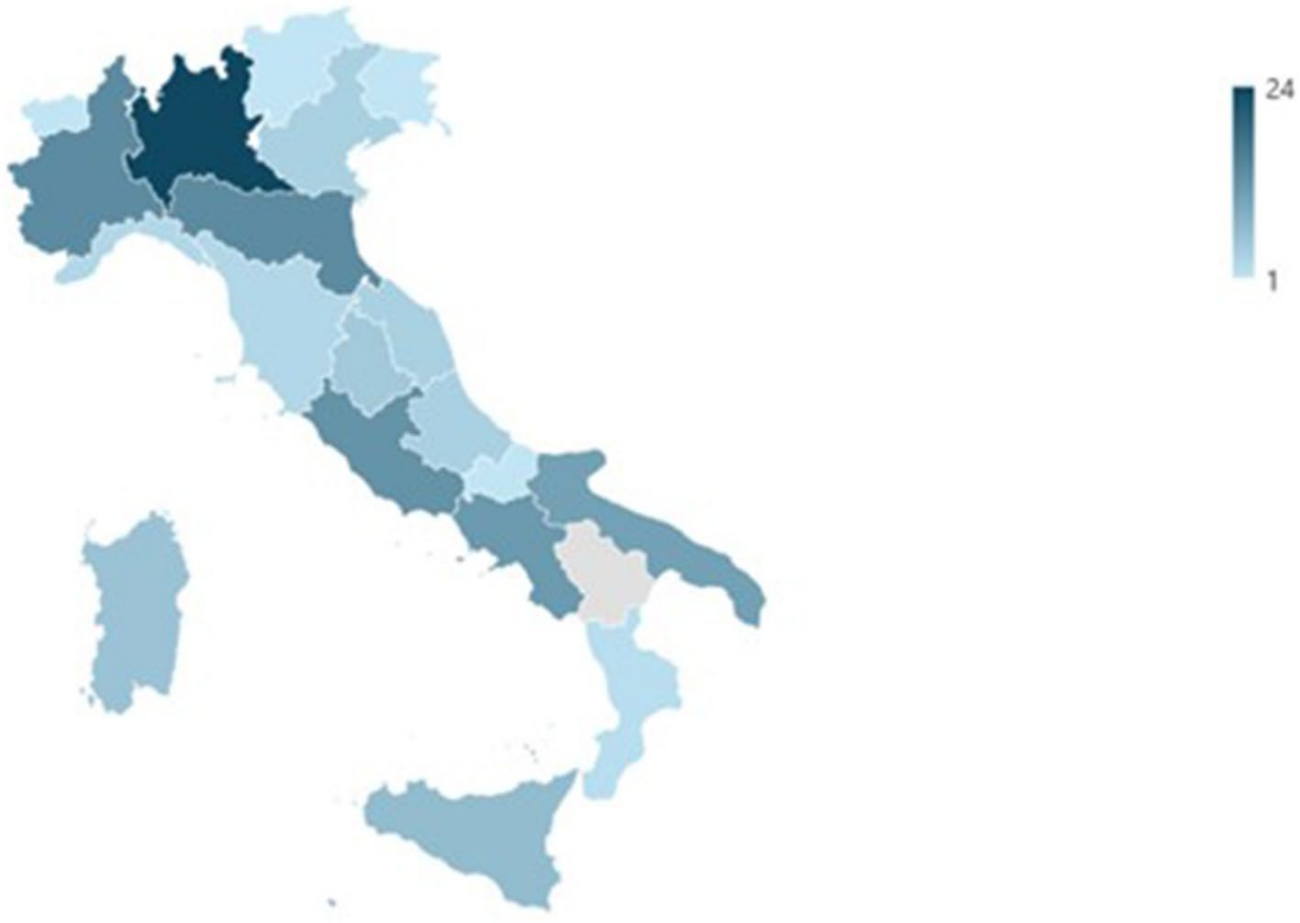
Regional distribution of responders to the survey

In Northern Italy (*n* = 62), the majority of centers are located in Lombardy (*n* = 24); in Central Italy (*n* = 31), most are in Lazio (*n* = 13); and in Southern Italy (*n* = 37), most centers are in Campania (*n* = 13) (Fig. 1) [17].

### Telemedicine for pain management

**Fig. 2:**
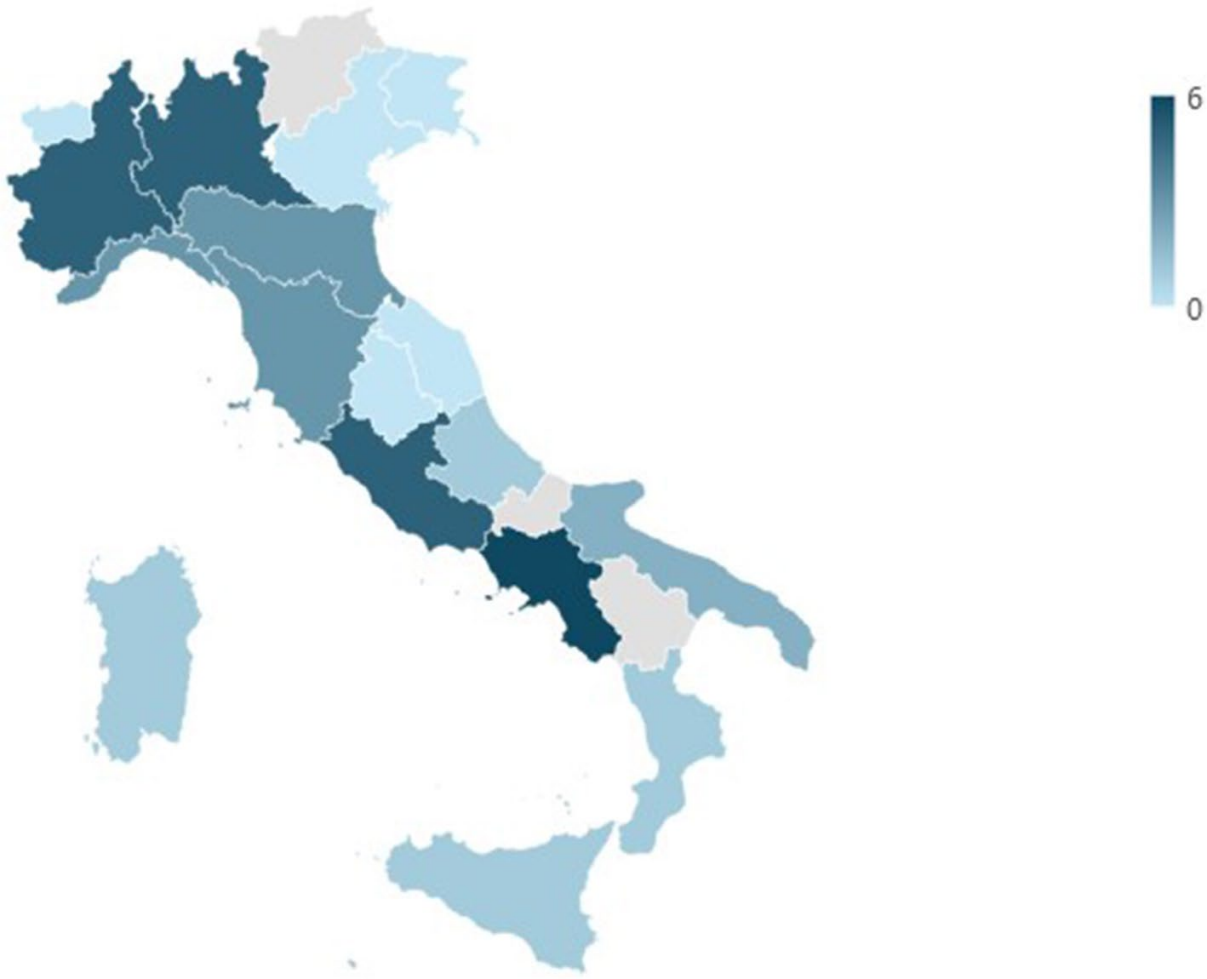
Regional distribution of telemedicine-based pain management centers

Telemedicine was routinely used in 40% of the responding centers (*n* = 38), mostly in Campania (*n* = 6), Lombardy (*n* = 5), Lazio (*n* = 5), and Piedmont (*n* = 5) (Fig. 2). In 61.2% of cases, centers use telemedicine for various clinical purposes.

Among the centers not yet implementing telemedicine, 9.3% are in the process of completing the necessary steps, 51.9% are planning to start, and 38.9% have no interest in starting telemedicine.

The reasons for not using telemedicine include the absence of protocols (16.7%), lack of resources (44.4%), bureaucratic obstacles (25%), and other reasons (19%).

The timeline for starting telemedicine varied as 21.1% of the centers began before the COVID-19 pandemic, 36.8% during the pandemic, and 42.1% after the pandemic.

### Telemedicine care pathway

Among the centers conducting telemedicine, 47.4% have a protocol for telemedicine use within the care pathway. The first visit was conducted in person in 85.9% of the centers. Patient selection criteria for telemedicine are absent in 28.9% of the centers, whereas 71.1% had established criteria. Additionally, 81.6% of the centers have criteria for returning patients to in-person visits.

In terms of operational aspects, 57.9% of the centers had scheduled days for telemedicine visits, with half of these having more than 1 day per week (27.8% 2 days a week, 22.2% more 3 or more days a week). The remaining 42.1% conducted telemedicine activities as needed.

**Fig. 3:**
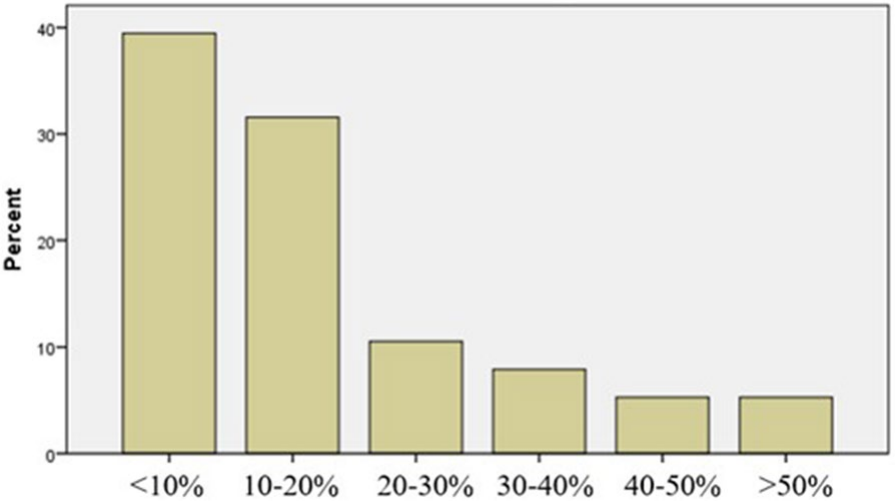
Impact of telemedicine use on service delivery in pain medicine

The impact of telemedicine on pain therapy services showed an increase in overall services, with 39.5% of the centers reporting an increase of less than 10% and 5.3% reporting an increase of more than 50% (Fig. 3).

Technological capabilities were also assessed, with 72.8% of the centers able to share images such as computerized tomography (CT) and computerized tomography (MRI) scans between patients and doctors during teleconsultations. However, only 9.1% of the centers had telemedicine software that utilized artificial intelligence strategies (e.g., for telemonitoring and alert systems).

Teleconsultation capabilities were present in 39.4% of the centers, with teleconsultations possible with neurologists, orthopedists, general practitioners, and other specialists (15.4% each).

Additionally, 33.6% of the centers conducted telemedicine for fragile patients (such as pediatric patients and those with cognitive disabilities), with 54.4% having a dedicated path for these patients.

Finally, 21.2% of the centers could connect hub/spoke centers through teleconsultation.

## Discussion

The findings of this survey underscore the significant yet varied adoption of telemedicine in pain management across Italy. The data reveal a considerable shift towards integrating telemedicine into clinical practice, reflecting broader trends in healthcare delivery transformation driven by technological advancements and the exigencies imposed by the COVID-19 pandemic [18–22].

The survey highlighted that 40% of the responding centers are routinely using telemedicine for pain management, indicating a solid foothold for telemedicine in this domain. However, the regional disparities in adoption are noteworthy. Northern Italy, particularly Lombardy, shows a higher concentration of telemedicine adoption, which could be attributed to better infrastructure and resource availability [23, 24]. In contrast, the uptake in southern and central regions, while significant, appears more fragmented [16]. This disparity underscores the need for targeted strategies to address regional inequities and ensure a more uniform adoption of telemedicine services across the country [25–27].

Despite the promising integration of telemedicine, several barriers impede its broader adoption [2, 28, 29]. A substantial proportion of centers (38.9%) expressed no interest in starting telemedicine, often citing the absence of protocols (16.7%), lack of resources (44.4%), and bureaucratic obstacles (25%). These challenges highlight systemic issues that require comprehensive policy interventions and resource allocation to overcome [30]. The creation of standardized protocols and streamlined bureaucratic processes, coupled with investment in necessary technological infrastructure, could significantly lower these barriers [31].

The operational dynamics of telemedicine use in pain management reveal a structured yet flexible approach. Over half of the centers (57.9%) have scheduled telemedicine visits, with a significant portion offering these services multiple days a week. This structured approach indicates a commitment to integrating telemedicine into routine care. However, the fact that 42.1% of centers operate telemedicine on an as-needed basis suggests variability in demand and resource allocation.

Moreover, the presence of protocols in 47.4% of the centers and patient selection criteria in 71.1% indicate an emerging framework for telemedicine use. However, the absence of such criteria in a notable percentage of centers suggests room for improvement in standardizing telemedicine practices. Establishing clear guidelines and best practices could enhance the consistency and quality of telemedicine services [32].

The impact of telemedicine on service delivery is generally positive, with many centers reporting increases in overall service volume. This trend reflects the potential of telemedicine to enhance access to care, particularly for chronic pain patients who may face mobility issues or reside in remote areas [23, 33]. The reported increases in service volume, though varied, suggest that telemedicine can significantly augment traditional care pathways and optimize healthcare strategies, for example, for addressing the issue of opioid management [34].

Technological capabilities play a crucial role in the effective implementation of telemedicine. The ability to share diagnostic images during teleconsultations in 72.8% of the centers is a positive development, facilitating comprehensive remote assessments. However, the limited use of advanced telemedicine software with artificial intelligence (AI) (9.1%) indicates an area ripe for development. The integration of AI could enhance telemonitoring and alert systems, providing more proactive and personalized care [7, 35]. Notably, by implementing AI strategies, telemedicine platforms can analyze vast amounts of patient data in real time, enabling more accurate and timely assessments of pain management needs. This capability can also be useful to facilitate the development of sophisticated telemonitoring systems that continuously track patient progress and detect subtle changes in health status [36]. Additionally, AI-driven alert systems can promptly notify healthcare providers of potential issues or deterioration in patient conditions, allowing for swift intervention and adjustment of treatment plans. Moreover, the predictive capabilities of AI can aid in anticipating future pain management needs based on individual patient profiles, enabling the implementation of proactive and personalized care strategies tailored to each patient’s unique circumstances [37–40].

The capability to conduct teleconsultations with various specialists in 39.4% of the centers underscores the interdisciplinary potential of telemedicine. Furthermore, the provision of telemedicine services for fragile patients, such as pediatric patients, in 33.6% of the centers, with dedicated pathways in over half of these, highlights the inclusivity of telemedicine initiatives. This focus on vulnerable populations ensures that telemedicine can cater to diverse patient needs, enhancing overall healthcare equity [41, 42].

## Conclusions

The survey conducted by SIAARTI provides valuable insights into the current state of telemedicine in pain management in Italy. While the adoption of telemedicine shows promising trends, addressing the highlighted barriers and disparities is crucial for optimizing its use. Future strategies should focus on standardizing protocols, enhancing technological infrastructure, and ensuring equitable access across regions. By addressing these challenges, telemedicine can fully realize its potential to transform pain management and broader healthcare delivery.

## Data Availability

The data that support the findings of this study are not openly available due to reasons of sensitivity and are available from the corresponding author upon reasonable request.

## Abbreviations

AI: Artificial intelligence
CT: Computerized tomography
MRI: Magnetic resonance imaging
SIAARTI: Italian Society of Analgesia, Anesthesia, Resuscitation, and Intensive Care
